# Biphenyl-4-carbaldehyde azine

**DOI:** 10.1107/S1600536808038622

**Published:** 2008-11-26

**Authors:** Wagee A. Yehye, Azhar Ariffin, Noorsaadah A. Rahman, Seik Weng Ng

**Affiliations:** a Department of Chemistry, University of Malaya, 50603 Kuala Lumpur, Malaysia

## Abstract

The complete mol­ecule of the title compound, C_26_H_20_N_2_, is generated by crystallographic inversion symmetry. The terminal phenyl ring is twisted by 19.2 (1)° with respect to the adjacent phenyl­ene ring.

## Related literature

For the synthesis, see: Malkes & Timchenko (1961 ▶). For biological evaluation, see: Cremlyn *et al.* (1991 ▶). The compound is a formylating agent for aromatic compounds; see: Kantlehner *et al.* (2004 ▶). When treated with cerium ammonium nitrate, the aldehyde is regenerated; see Giurg & Mlochowski (1999 ▶).
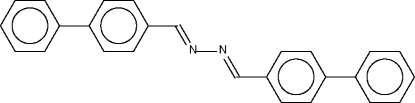


## Experimental

### 

#### Crystal data

C_26_H_20_N_2_
*M* *_r_* = 360.44
Monoclinic, 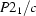

*a* = 20.5417 (6) Å
*b* = 7.1358 (2) Å
*c* = 6.3402 (2) Å
β = 93.632 (2)°
*V* = 927.49 (5) Å^3^
*Z* = 2
Mo *K*α radiation
μ = 0.08 mm^−1^
*T* = 100 (2) K
0.40 × 0.25 × 0.10 mm

#### Data collection

Bruker SMART APEX CCD diffractometer
Absorption correction: none
6044 measured reflections
2104 independent reflections
1607 reflections with *I* > 2σ(*I*)
*R*_int_ = 0.025

#### Refinement

*R*[*F* ^2^ > 2σ(*F* ^2^)] = 0.042
*wR*(*F* ^2^) = 0.127
*S* = 1.05
2104 reflections
127 parameters
H-atom parameters constrained
Δρ_max_ = 0.31 e Å^−3^
Δρ_min_ = −0.21 e Å^−3^

### 

Data collection: *APEX2* (Bruker, 2007 ▶); cell refinement: *SAINT* (Bruker, 2007 ▶); data reduction: *SAINT*; program(s) used to solve structure: *SHELXS97* (Sheldrick, 2008 ▶); program(s) used to refine structure: *SHELXL97* (Sheldrick, 2008 ▶); molecular graphics: *X-SEED* (Barbour, 2001 ▶); software used to prepare material for publication: *publCIF* (Westrip, 2008 ▶).

## Supplementary Material

Crystal structure: contains datablocks global, I. DOI: 10.1107/S1600536808038622/hb2856sup1.cif

Structure factors: contains datablocks I. DOI: 10.1107/S1600536808038622/hb2856Isup2.hkl

Additional supplementary materials:  crystallographic information; 3D view; checkCIF report

## Figures and Tables

**Fig. 1.**
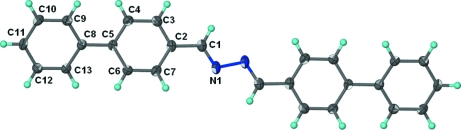
The molecular structure of (I) with atoms shown at the 70% probability level. Hydrogen atoms are drawn as spheres of arbitrary radiius. The unlabelled atoms are generated by the symmetry operation (1–x, 1–y, 1–z).

## supplementary crystallographic information

### Comment

The complete molecule of the title compound, (I) is generated by crystallographic inversion symmetry (Fig. 1). The terminal phenyl ring is twisted by 19.2 (1) ° with respect to the phenylene ring.

### Experimental

4-Phenyl benzaldehyde (0.72 g, 4 mmol) and 80% hydrazine hydrate (0.10 g, 2 mmol) were heated in ethanol (25 ml) for 1 h. The resulting product was filtered and washed with ethanol and then recrystallized from hexane to yield yellow prisms of (I).

### Refinement

The H atoms were placed in calculated positions (C—H = 0.95 Å) and refined as riding with *U*(H) = 1.2*U*(C).

### Crystal data

**Table:**

C_26_H_20_N_2_	*F*_000_ = 380
*M**_r_* = 360.44	*D*_x_ = 1.291 Mg m^−^^3^
Monoclinic, *P*2_1_/*c*	Mo *K*α radiation λ = 0.71073 Å
Hall symbol: -P 2ybc	Cell parameters from 1869 reflections
*a* = 20.5417 (6) Å	θ = 2.9–26.2º
*b* = 7.1358 (2) Å	µ = 0.08 mm^−^^1^
*c* = 6.3402 (2) Å	*T* = 100 (2) K
β = 93.632 (2)º	Prism, yellow
*V* = 927.49 (5) Å^3^	0.40 × 0.25 × 0.10 mm
*Z* = 2	

### Data collection

**Table:**

Bruker SMART APEX CCD diffractometer	1607 reflections with *I* > 2σ(*I*)
Radiation source: fine-focus sealed tube	*R*_int_ = 0.025
Monochromator: graphite	θ_max_ = 27.5º
*T* = 100(2) K	θ_min_ = 1.0º
ω scans	*h* = −25→26
Absorption correction: None	*k* = −8→9
6044 measured reflections	*l* = −8→8
2104 independent reflections	

### Refinement

**Table:**

Refinement on *F*^2^	Secondary atom site location: difference Fourier map
Least-squares matrix: full	Hydrogen site location: inferred from neighbouring sites
*R*[*F*^2^ > 2σ(*F*^2^)] = 0.042	H-atom parameters constrained
*wR*(*F*^2^) = 0.127	*w* = 1/[σ^2^(*F*_o_^2^) + (0.0641*P*)^2^ + 0.2493*P*] where *P* = (*F*_o_^2^ + 2*F*_c_^2^)/3
*S* = 1.06	(Δ/σ)_max_ = 0.001
2104 reflections	Δρ_max_ = 0.31 e Å^−^^3^
127 parameters	Δρ_min_ = −0.21 e Å^−^^3^
Primary atom site location: structure-invariant direct methods	Extinction correction: none

### Special details

**Table:**

Geometry. All e.s.d.'s (except the e.s.d. in the dihedral angle between two l.s. planes) are estimated using the full covariance matrix. The cell e.s.d.'s are taken into account individually in the estimation of e.s.d.'s in distances, angles and torsion angles; correlations between e.s.d.'s in cell parameters are only used when they are defined by crystal symmetry. An approximate (isotropic) treatment of cell e.s.d.'s is used for estimating e.s.d.'s involving l.s. planes.

### Fractional atomic coordinates and isotropic or equivalent isotropic displacement parameters (Å^2^)

**Table:**

	*x*	*y*	*z*	*U*_iso_*/*U*_eq_	
N1	0.46654 (6)	0.48506 (18)	0.47390 (19)	0.0260 (3)	
C1	0.45139 (7)	0.5211 (2)	0.2795 (2)	0.0229 (3)	
H1	0.4847	0.5598	0.1916	0.027*	
C2	0.38464 (7)	0.50463 (19)	0.1880 (2)	0.0202 (3)	
C3	0.37032 (7)	0.5594 (2)	−0.0206 (2)	0.0218 (3)	
H3	0.4045	0.6006	−0.1034	0.026*	
C4	0.30685 (7)	0.5548 (2)	−0.1095 (2)	0.0205 (3)	
H4	0.2983	0.5927	−0.2522	0.025*	
C5	0.25534 (6)	0.49541 (19)	0.0078 (2)	0.0169 (3)	
C6	0.27039 (7)	0.43846 (19)	0.2177 (2)	0.0198 (3)	
H6	0.2363	0.3972	0.3008	0.024*	
C7	0.33352 (7)	0.4412 (2)	0.3054 (2)	0.0212 (3)	
H7	0.3424	0.3998	0.4467	0.025*	
C8	0.18693 (6)	0.49529 (18)	−0.08434 (19)	0.0170 (3)	
C9	0.16848 (7)	0.60293 (19)	−0.2631 (2)	0.0202 (3)	
H9	0.2005	0.6745	−0.3287	0.024*	
C10	0.10445 (7)	0.6072 (2)	−0.3461 (2)	0.0215 (3)	
H10	0.0932	0.6811	−0.4676	0.026*	
C11	0.05681 (7)	0.5041 (2)	−0.2529 (2)	0.0200 (3)	
H11	0.0128	0.5083	−0.3084	0.024*	
C12	0.07425 (6)	0.39455 (19)	−0.0772 (2)	0.0197 (3)	
H12	0.0420	0.3224	−0.0132	0.024*	
C13	0.13839 (6)	0.38970 (19)	0.0054 (2)	0.0186 (3)	
H13	0.1496	0.3133	0.1249	0.022*	

### Atomic displacement parameters (Å^2^)

**Table:**

	*U*^11^	*U*^22^	*U*^33^	*U*^12^	*U*^13^	*U*^23^
N1	0.0154 (6)	0.0334 (7)	0.0285 (7)	−0.0007 (5)	−0.0034 (5)	−0.0004 (5)
C1	0.0174 (7)	0.0249 (8)	0.0261 (7)	0.0009 (5)	−0.0003 (5)	−0.0009 (6)
C2	0.0171 (7)	0.0195 (7)	0.0235 (7)	0.0013 (5)	−0.0018 (5)	−0.0020 (5)
C3	0.0181 (7)	0.0244 (7)	0.0231 (7)	−0.0002 (5)	0.0028 (5)	0.0015 (5)
C4	0.0205 (7)	0.0229 (7)	0.0178 (6)	0.0009 (5)	0.0002 (5)	0.0015 (5)
C5	0.0168 (7)	0.0149 (6)	0.0187 (6)	0.0012 (5)	−0.0017 (5)	−0.0015 (5)
C6	0.0188 (7)	0.0209 (7)	0.0196 (6)	−0.0007 (5)	0.0013 (5)	0.0015 (5)
C7	0.0221 (7)	0.0225 (7)	0.0186 (6)	0.0005 (5)	−0.0020 (5)	0.0007 (5)
C8	0.0185 (7)	0.0164 (6)	0.0160 (6)	0.0012 (5)	−0.0010 (5)	−0.0028 (5)
C9	0.0203 (7)	0.0201 (7)	0.0200 (6)	−0.0024 (5)	0.0000 (5)	0.0025 (5)
C10	0.0245 (7)	0.0213 (7)	0.0182 (6)	0.0011 (5)	−0.0036 (5)	0.0020 (5)
C11	0.0170 (7)	0.0227 (7)	0.0197 (6)	0.0018 (5)	−0.0037 (5)	−0.0036 (5)
C12	0.0185 (7)	0.0210 (7)	0.0196 (6)	−0.0016 (5)	0.0022 (5)	−0.0010 (5)
C13	0.0195 (7)	0.0191 (7)	0.0169 (6)	0.0008 (5)	−0.0004 (5)	0.0010 (5)

### Geometric parameters (Å, °)

**Table:**

N1—C1	1.2784 (19)	C6—H6	0.9500
N1—N1^i^	1.410 (2)	C7—H7	0.9500
C1—C2	1.4592 (18)	C8—C13	1.3989 (18)
C1—H1	0.9500	C8—C9	1.4012 (18)
C2—C3	1.3927 (18)	C9—C10	1.3858 (19)
C2—C7	1.4006 (19)	C9—H9	0.9500
C3—C4	1.3874 (18)	C10—C11	1.3863 (19)
C3—H3	0.9500	C10—H10	0.9500
C4—C5	1.3969 (19)	C11—C12	1.3888 (19)
C4—H4	0.9500	C11—H11	0.9500
C5—C6	1.4073 (18)	C12—C13	1.3870 (18)
C5—C8	1.4873 (17)	C12—H12	0.9500
C6—C7	1.3784 (18)	C13—H13	0.9500
			
C1—N1—N1^i^	111.73 (15)	C6—C7—H7	119.7
N1—C1—C2	122.17 (13)	C2—C7—H7	119.7
N1—C1—H1	118.9	C13—C8—C9	117.44 (12)
C2—C1—H1	118.9	C13—C8—C5	121.38 (11)
C3—C2—C7	118.38 (12)	C9—C8—C5	121.17 (12)
C3—C2—C1	119.45 (13)	C10—C9—C8	121.38 (12)
C7—C2—C1	122.14 (12)	C10—C9—H9	119.3
C4—C3—C2	121.03 (12)	C8—C9—H9	119.3
C4—C3—H3	119.5	C9—C10—C11	120.34 (12)
C2—C3—H3	119.5	C9—C10—H10	119.8
C3—C4—C5	121.01 (12)	C11—C10—H10	119.8
C3—C4—H4	119.5	C10—C11—C12	119.17 (12)
C5—C4—H4	119.5	C10—C11—H11	120.4
C4—C5—C6	117.55 (12)	C12—C11—H11	120.4
C4—C5—C8	121.34 (11)	C13—C12—C11	120.49 (13)
C6—C5—C8	121.11 (12)	C13—C12—H12	119.8
C7—C6—C5	121.48 (12)	C11—C12—H12	119.8
C7—C6—H6	119.3	C12—C13—C8	121.16 (12)
C5—C6—H6	119.3	C12—C13—H13	119.4
C6—C7—C2	120.53 (12)	C8—C13—H13	119.4
			
N1^i^—N1—C1—C2	−178.70 (14)	C4—C5—C8—C13	161.70 (13)
N1—C1—C2—C3	175.14 (14)	C6—C5—C8—C13	−19.36 (19)
N1—C1—C2—C7	−2.6 (2)	C4—C5—C8—C9	−19.07 (19)
C7—C2—C3—C4	1.2 (2)	C6—C5—C8—C9	159.86 (13)
C1—C2—C3—C4	−176.66 (13)	C13—C8—C9—C10	0.99 (19)
C2—C3—C4—C5	0.1 (2)	C5—C8—C9—C10	−178.27 (12)
C3—C4—C5—C6	−0.8 (2)	C8—C9—C10—C11	0.1 (2)
C3—C4—C5—C8	178.21 (13)	C9—C10—C11—C12	−0.9 (2)
C4—C5—C6—C7	0.1 (2)	C10—C11—C12—C13	0.7 (2)
C8—C5—C6—C7	−178.86 (12)	C11—C12—C13—C8	0.5 (2)
C5—C6—C7—C2	1.2 (2)	C9—C8—C13—C12	−1.26 (19)
C3—C2—C7—C6	−1.8 (2)	C5—C8—C13—C12	177.99 (12)
C1—C2—C7—C6	175.96 (13)		

Symmetry codes: (i) −*x*+1, −*y*+1, −*z*+1.
